# Multi-pinhole collimator design in different numbers of projections for brain SPECT

**DOI:** 10.3389/fmed.2023.1211726

**Published:** 2023-09-28

**Authors:** Wenbo Huang, Greta S. P. Mok

**Affiliations:** ^1^Biomedical Imaging Laboratory (BIG), Department of Electrical and Computer Engineering, Faculty of Science and Technology, University of Macau, Taipa, Macau SAR, China; ^2^Center for Cognitive and Brain Sciences, Institute of Collaborative Innovation, University of Macau, Taipa, Macau SAR, China

**Keywords:** multi-pinhole collimator, brain SPECT, Alzheimer's disease, Parkinson's disease, angular sampling

## Abstract

**Purpose:**

High resolution and sensitivity brain SPECT is promising for the accurate diagnosis of Alzheimer's disease (AD) and Parkinson's disease (PD). Multi-pinhole (MPH) collimators with a good performance in imaging small field-of-view (FOV) could be better used for brain SPECT. In this study, we aim to evaluate the impact of varying the number of pinholes and the number of projections on the performance of MPH brain SPECT.

**Methods:**

The system design was based on a commercial clinical dual-head SPECT/CT scanner, with target spatial resolutions of 12 mm and 8 mm for AD and PD SPECT, respectively. In total, 1–25 pinholes were modeled for 64, 32, 16, 8, 4, and 2 projections. The 3D NURBS-based HUman Brain (NHUB) phantom was used in the analytical simulation to model ^99m^Tc-HMPAO and ^99m^Tc-TRODAT distributions. The 2D Derenzo hot-rod phantom and star phantom were used in Monte Carlo simulations to evaluate the spatial resolution and angular sampling performance of MPH. The influence of different detector positions was also evaluated for 2, 4, and 6 angular views. The projections were reconstructed using the 3D MPH ML-EM method. Normalized mean square error, coefficient of variation, and image profiles were evaluated.

**Results:**

Along with the decrease in the number of projections, more pinholes are required to achieve the optimum performance. For 32 projections, 9- and 7-pinhole collimators provide the best normalized mean square error (NMSE) to the coefficient of variation (COV) trade-off for ^99m^Tc-HMPAO and ^99m^Tc-TRODAT, respectively. Detector positions substantially affect the image quality for MPH SPECT for 2 and 4 angular views. The smallest rod size for the Derenzo hot-rod phantom, which could be resolved, is 7.9 mm for the MPH general purpose collimator (MPGP) with more than 16 projections and 6.4 mm for MPH high-resolution collimator (MPHR) with more than 8 projections.

**Conclusion:**

The number of pinholes affects the performance of the MPH collimator, especially when the projection views become fewer. More pinholes are required for fewer projections to provide better angular sampling in MPH for complex activity distributions. Detector positions affect the image quality of MPH SPECT for 2 and 4 angular views, where L-mode acquisition is slightly superior to H-mode. MPH collimators exhibited improved spatial resolution and angular sampling compared with both LEHR and single pinhole collimators.

## 1. Introduction

With the increase in the aging population worldwide, neurodegenerative diseases such as Alzheimer's disease (AD) and Parkinson's disease (PD) pose a substantial burden in society. Fifty million people are estimated to suffer from dementia throughout the world in 2018, and this number is expected to triple by 2050 (1). On the other hand, in 2016, ~6.1 million individuals suffered from PD globally, and it caused 3.2 million disability-adjusted life years and 211,296 deaths (2). Currently, the number of drugs available for the treatment of Alzheimer's disease and Parkinson's disease is limited (3, 4), Lecanemab-irmb (5) and aducanumab (6) are currently the only two drugs approved by the US Food and Drug Administration (FDA) for AD treatment, both work by reducing the deposition of amyloid beta plaque. Levodopa (7) is the most effective medication for Parkinson's disease. Deep brain stimulation (8), which involves the surgical implantation of electrodes into a specific area of the brain, is another effective method for controlling symptoms. However, despite these available treatment options, the diseases cannot be cured yet. Early intervention remains the most effective treatment approach for both conditions. Thus, early diagnosis for delayed disease progression is increasingly important for these two diseases.

Along with the development of different tracers that can target beta-amyloid (9), tau protein (10), synaptic vesicle glycoprotein 2A (SV2A) density (11), acetylcholine (12), brain perfusion (13), and dopaminergic system (14), the role of radionuclide imaging, including PET and SPECT, in neurodegenerative diseases is well recognized. Usually, highly demanding production and transportation processes are needed for PET, especially if short-lived isotopes are used, whereas the half-life of SPECT isotopes is longer. Thus, SPECT is generally more accessible and economical than PET for PD and AD diagnosis and treatment monitoring. Current brain SPECT tracers include Tc-99m-labeled hexamethyl propylene amine oxime (HMPAO) for cerebral blood flow, ^99m^Tc-TRODAT, and ^123^I-ioflupane for targeting dopamine transporters in the striatum region, which can detect the onset of the disease much earlier than the development of symptoms. ^99m^Tc-TRODAT is a more common DAT tracer in Asia as compared with ^123^I-ioflupane. The biodistributions of these two tracers are similar, but ^99m^Tc-TRODAT has a lower striatal uptake with less photon scattering and penetration (15), while ^123^I-ioflupane suffers from contamination of high-energy photons.

The sensitivity of SPECT is much lower than PET due to the physical collimation, which is required to determine the incident angle of the gamma photons. Ma et al. have proposed a novel self-collimating SPECT system as a potential solution (16, 17). However, as for now, collimators remain an essential component in clinical SPECT. For brain imaging, as the object is smaller than the detector field-of-view (FOV), better trade-off can be achieved by using converging collimators, e.g., fan beam, cone beam, or pinhole collimators as compared with conventional parallel-hole collimators. Multi-pinhole (MPH) collimators have been proposed to further improve the resolution and sensitivity trade-off and the sampling problem of single-pinhole collimators. When the pinholes are focused on a common volume, MPH collimators can obtain multiple projections at a single detector location to improve both angular and axial sampling (18). Another advantage of MPH collimators is that the collimator penetration is greatly reduced as compared with other collimators, particularly for tracers with down scattering from high-energy photons, e.g., ^123^I (19), a common radionuclide for PD tracer such as ^123^I-ioflupane.

The MPH reconstructed image quality, e.g., resolution, detection efficiency, and artifacts depends on the number of pinholes, placement of pinholes, magnification factor, projection overlapping, truncation, aperture size, and acceptance angle (18, 20–22). Recently, different types of MPH collimators for clinical brain SPECT imaging have been proposed, mainly for research applications (23–28). However, there are limited studies to investigate the designs of MPH collimators for brain SPECT (29, 30). Our previous study proposed an optimized MPH design by maximizing the sensitivity for a target spatial resolution for a single angular position of a flat panel detector (31), showing 268% sensitivity improvement as compared with a conventional parallel-hole collimator based on the same spatial resolution. Improved sampling and sensitivity in MPH SPECT imaging can potentially reduce the number of required projection views, and the optimal MPH design may be different for different numbers of projections and brain applications. Moreover, improved sensitivity may not necessarily be translated to better image quality. Our current study evaluated the MPH performance for various numbers of projections based on actual reconstructed image quality. We aimed to investigate the performance of MPH collimators with various pinhole configurations for a number of different projection views in MPH brain perfusion and DAT SPECT.

## 2. Materials and methods

### 2.1. System description

Our system design is based on a commercial clinical dual-head SPECT/CT scanner (Infinia Hawkeye 4, GE Healthcare, USA). The active detector area is 540 mm × 400 mm, with 9.525 mm NaI crystal and a 3.2 mm intrinsic resolution. The maximum system radius of the system is 344 mm, which is the distance from the center of the field of view (CFOV) to the surface of the detector. The minimum system radius is 292 mm, which is large enough to accommodate and clear the shoulders of the 95 percentile Chinese population based on the national standard GB/T 10000-1988 (32). Meanwhile, the minimum radius of rotation (ROR) is set to be 135 mm, which is large enough to provide a 200 mm FOV for MPH SPECT to cover heads of 95 percentile Chinese population based on the national standard GB/T 2428-1998 (33).

**Figure 1:**
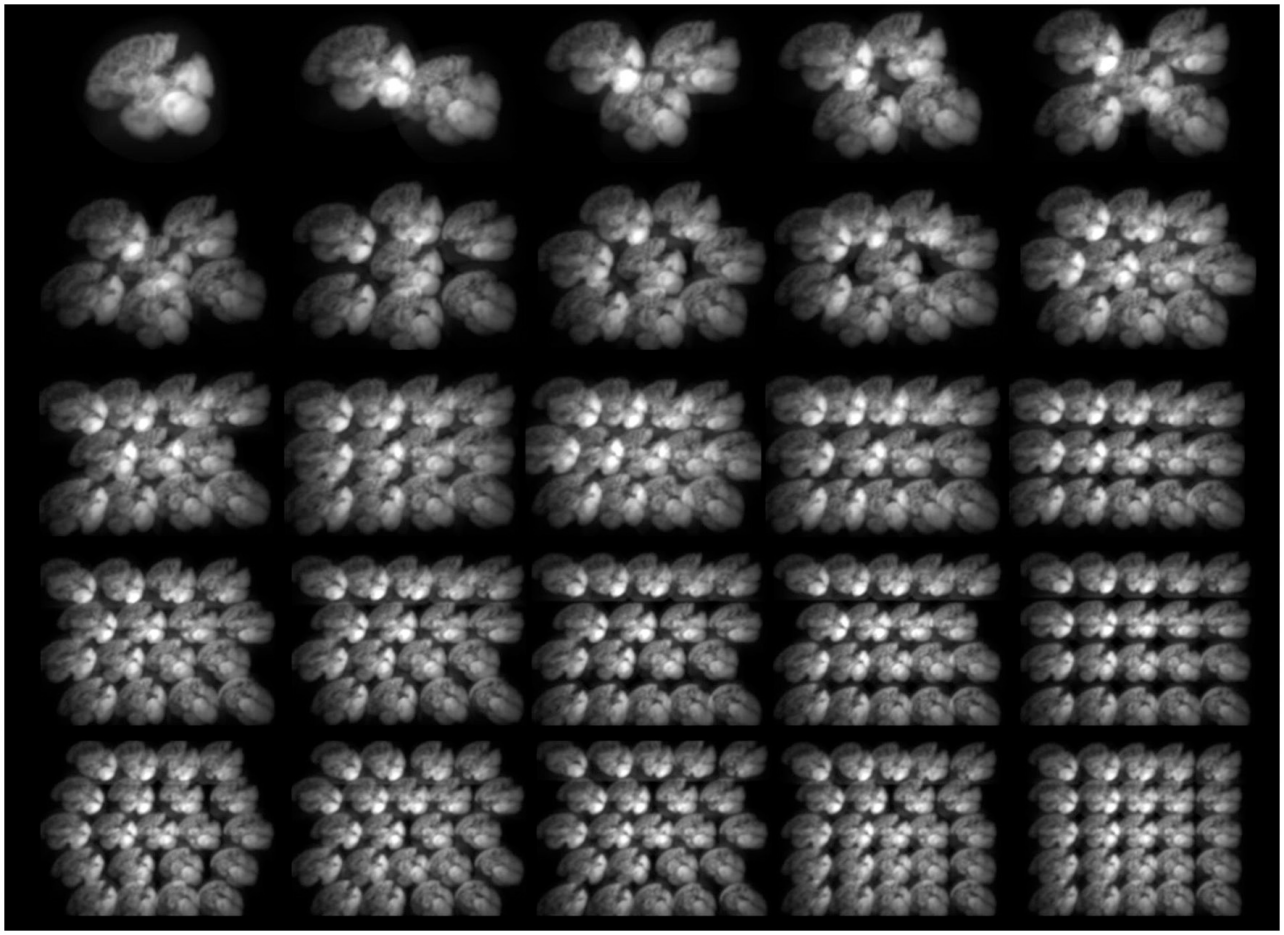
Sample noise-free projections of HMPAO generated by MPGP collimators with different numbers of pinholes (*n* = 1–25).

Two sets of MPH collimators for different target planar system spatial resolutions (*R*_*t*_): (1) 12 mm for AD (Multi-Pinhole General Purpose, MPGP); and (2) 8 mm for PD (Multi-Pinhole High Resolution, MPHR) are evaluated. The first one is the same system resolution for a low energy high resolution (LEHR) parallel-hole collimator at 225 mm ROR, a common setting for cerebral blood flow Tc-99m HMPAO SPECT. The second one is used for imaging the striatum region with a higher resolution requirement. The pinholes on the collimator are positioned with projections fully utilizing the coverage of the detector area, providing more number of angular and axial sampling, and keeping the total projection overlapping among all pinhole projections <20% (20, 34) (Figure 1). The pinhole arrangement is the same for MPGP and MPHR, while the pinhole aperture size is smaller for the latter. We model different numbers of projections (*n* = 2–64) and pinholes (*n* = 1–25), as well as ROR and corresponding collimator length for the respective target resolution. The specific design parameters for different MPH collimators are listed in Supplementary Table S1. In addition, as a dual-head SPECT scanner, two detectors can be configured as perpendicular (L-mode) or parallel (H-mode) to each other (35), with the latter being a more common setting in conventional brain SPECT using LEHR. In this study, we also evaluate various MPH performances for H- and L-modes with different detector positions for brain SPECT.

### 2.2. Phantoms

**Figure 2:**
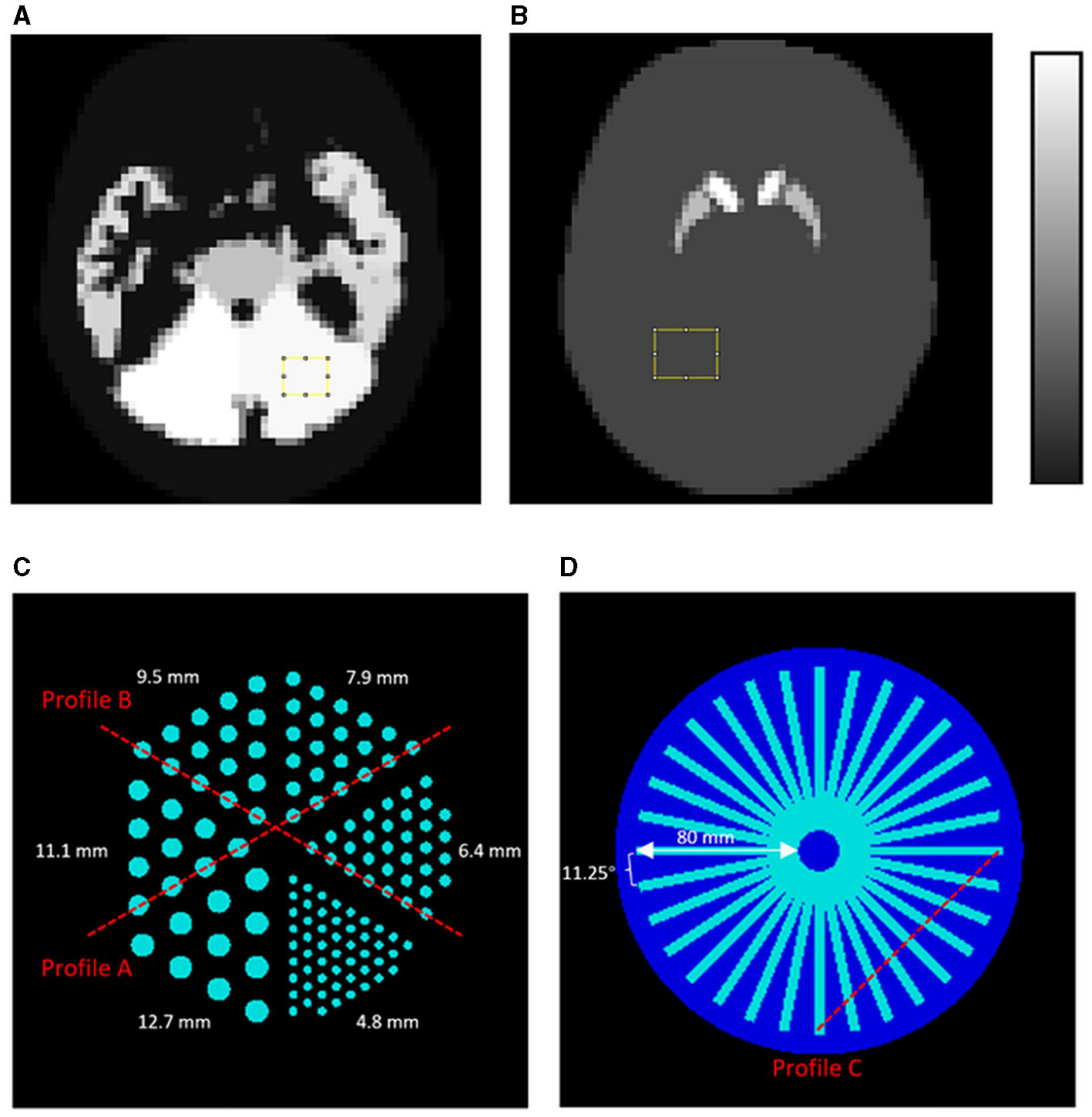
NHUB brain phantom with **(A)** ^99m^Tc-HMPAO distribution and **(B)** ^99m^Tc-TRODAT distribution. The uniform regions marked by the yellow squares are used for the noise analysis and the background region for calculating the SBR. Moreover, 2D phantoms used in the Monte Carlo simulations: **(C)** a Derenzo hot-rod phantom and **(D)** a cold star phantom made up of 32 columns. Profiles at different locations (red dashed lines) are assessed.

The NURBS-based HUman Brain (NHUB) phantom (36) with ^99m^Tc-HMPAO and ^99m^Tc-TRODAT distributions is modeled for AD and PD imaging, respectively. For HMPAO, the uptake ratios of various normal regions are set based on the study mentioned in the reference (37) (Figure 2A), e.g., gray to white matter is ~10:1, while the striatal binding ratio (SBR) is set to 9:1 (38) (Figure 2B) for normal TRODAT distributions. The voxel size and matrix size of the phantom are set to be 3.125 × 3.125 × 3.125 mm^3^ and 64 × 64 × 64, respectively.

We also simulate a 2D Derenzo phantom (Figure 2C) and a 2D star phantom (Figure 2D), with the former to demonstrate the system resolution while the latter to demonstrate the angular sampling of the proposed MPH collimators. Hot rods with diameters of 4.8 mm, 6.4 mm, 7.9 mm, 9.5 mm, 11.1 mm, and 12.7 mm are modeled in the Derenzo phantom, with rod center-to-center distance twice the rod diameter. The angular interval of each bar in the star phantom is 11.25°. Both phantoms are situated within a water-filled 2D circle container with a radius of 100 mm.

### 2.3. Analytical simulation

To evaluate the performance of the proposed MPH collimator designs, we first perform analytical simulations based on a 3D analytical MPH projector (39), without modeling attenuation, scatter, and penetration, assuming perfect corrections.

To determine the optimal number of pinholes for different projection views, we use the NHUB phantom to generate 64, 32, 16, 8, 4, and 2 noise-free projections over 360° based on H-mode acquisition. For each number of projections, we model 1–25 pinholes for HMPAO and 1–20 pinholes for TRODAT. Then, the noise-free projections generated from both MPGP and MPHR collimators are scaled using the count level of clinical LEHR SPECT projection data as reference (*C*_*ref*_) (Eq. 1).


(1) $$\begin{array}{l} \;C_{s}=\frac{C_{ref}\times n_{p}\times S_{MPH}}{n_{ref}\times S_{LEHR}} \end{array}$$

where *C*_*s*_ is the total count of scaled noise-free projections, *n*_*p*_ is the number of simulated projection views, *S*_*MPH*_ is the sensitivity of the MPH collimator with different numbers of pinholes based on previous studies (31), *n*_*ref*_ is 120 which represents the number of projection views in the referenced clinical data, and *S*_*LEHR*_ is the sensitivity of LEHR collimator which is 0.01%. For ^99m^Tc-HMPAO, 1,000 MBq injection activity (~5 M projection counts) is set based on The European Association of Nuclear Medicine (EANM) guidelines (40). For ^99m^Tc-TRODAT, the referenced clinical data were based on a 740 MBq injection activity (~3 million projection counts). Poisson noise is, then, added to the scaled projections, with a mean equal to variance to simulate realistic noise levels.

**Figure 3:**
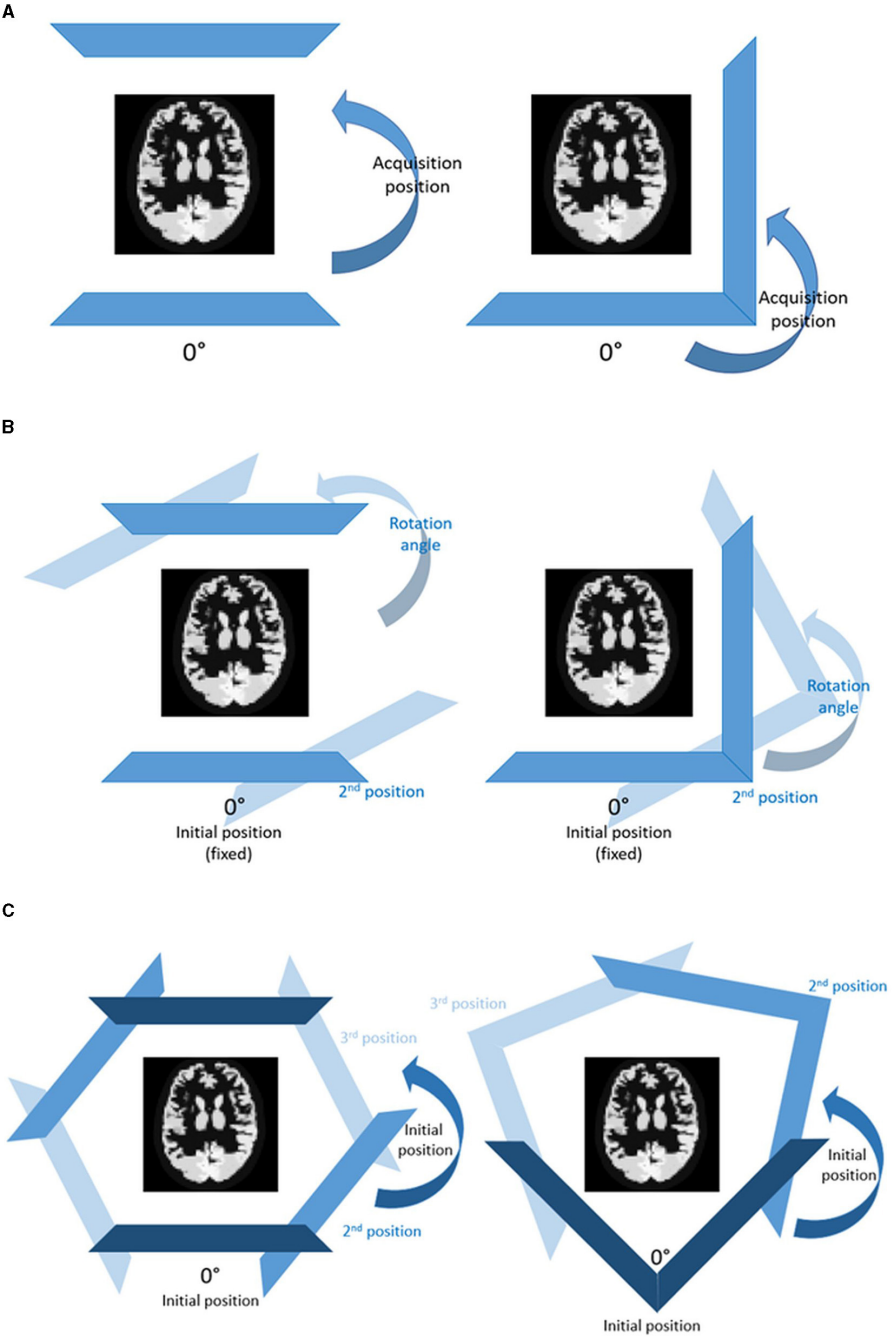
H-mode (left) and L-mode (right) configurations for **(A)** 2, **(B)** 4, and **(C)** 6 angular views on both HMPAO and TRODAT distributions using analytical simulations.

When projection views are limited, positions of the angular sampling may have a more significant effect on reconstructed image quality. To evaluate this effect, noise-free projections with 2, 4, and 6 views are generated based on H-mode and L-mode with different detector positions. For 2 views, both H- and L-modes are simulated by setting the acquisition position counter-clockwise from 0° to 360°, each with a 20° increment (Figure 3A). For four projections, we simulate H- and L-mode acquisitions with one angular rotation, using the 0°/180° for H-mode and 0°/90°for L-mode as initial positions and set the rotation angle from 0° to 180° to the 2nd position, each with a 20° increment (Figure 3B). The initial positions are chosen based on the two view results. For 6 projections, we set different initial positions ranging from 0° to 50°, with 10° increments. We then model 2 more pairs of detector positions spaced averagely over 360° for both H-mode and L-mode, resulting in each position being spaced 60° apart for H-mode and 120° apart for L-mode (Figure 3C). Supplementary Table S2 shows the detector position configurations for 2, 4, and 6 angular views for both HMPAO and TRODAT distributions. We simulate 9-pinhole and 15-pinhole collimators as they have similarly high sensitivity based on our previous studies (31), to investigate if different numbers of pinholes affect the choice of angular positions.

### 2.4. Monte Carlo simulation

To independently assess collimator performance concerning system resolution and angular sampling, regardless of projection overlapping, truncation, and noise effects, we conducted simulations employing the 2D Derenzo phantom and 2D star phantom through Geant4 Application for Tomographic Emission (GATE) v8.2 (41). GATE provided more realistic evaluations of our proposed MPGP and MPHR collimators, modeling all physical image degradation factors. To compare with the MPH collimator, we also model the LEHR collimator. The MPH collimators are modeled using a tungsten plate measuring 540 mm × 400 mm × 6 mm, while the LEHR collimator is modeled using a lead plate measuring 540 mm × 400 mm × 35 mm (septal length). Pinholes on the collimators are modeled by using two cones with overlapping tips and setting the material to be air. MGHR with smaller pinholes can be modeled by setting two cones further away from each other. In the case of LEHR, regular hexagonal holes were modeled with a side length of 1.5 mm. Other components of the SPECT detector are also modeled, including NaI crystal, lead shielding around the collimator and crystal, and the glass back compartment behind the crystal. All physical image degradation factors, including attenuation, scatter, penetration, and backscatter, are modeled during simulations. To mimic an almost noise-free scenario, we simulated 8 M photons in GATE with no obvious intensity fluctuations observed in the image profiles. We evaluate various configurations of pinholes, including 1, 3, 5, 9, and 14 pinholes, in combination with different numbers of projections: 64, 32, 16, 8, and 4. The acquisition time during the simulation is set to be 1200 s over 360° for all acquisition strategies. As MPGP and MPHR have the same pinhole arrangement, the angular sampling was only evaluated on MPGP using the 2D star phantom.

### 2.5. Reconstruction

A 3D MPH Maximum-Likelihood Expectation-Maximization (ML-EM) algorithm is used to reconstruct the noise-free projections with up to 1,000 iterations for HMPAO and 100 iterations for TRODAT. The noisy projections are reconstructed with up to 80 iterations for HMPAO and 40 iterations for TRODAT. The 2D Derenzo and star phantom are both reconstructed with up to 40 iterations without scatter and attenuation correction. The voxel size and matrix size of reconstructed images are set to be 3.125 mm and 200 × 200 × 200, respectively, which is large enough to avoid truncation artifacts. Afterward, the reconstructed images are extracted back to the original phantom size, i.e., 64 × 64 × 64 for analytical simulation and 64 × 64 × 1 for Monte Carlo simulation for further analysis.

### 2.6. Data analysis

Normalized mean square error (NMSE) is evaluated for both noise-free and noisy reconstructed images (Eq. 2). For HMPAO, it is calculated in the whole brain region, while for TRODAT, it is specifically calculated in the striatum region (29 × 23 × 17 voxels for 91 × 72 × 53 mm3).


(2) $$\;NMSE=\frac{\sum_{i=1}^{N}{\left(x_{i}-\lambda_{i}\right)}^{2}}{\sum_{i=1}^{N}\lambda_{i}{}^{2}}$$

where *N* is the total number of voxels, λ is the voxel value in the original phantom, *x* is the voxel value in reconstructed images, and *i* is the voxel index.

The SBR is calculated for TRODAT based on Eq. 3 as follows:


(3) $$\begin{array}{l} \;SBR=\frac{{\bar{\lambda }}_{s}}{{\bar{\lambda }}_{B}}-1 \end{array}$$

where ${\bar{\lambda }}_{s}$ is the mean value of the striatum region, and ${\bar{\lambda }}_{B}$ is the mean value of the background region. The striatum region is obtained by applying the known striatum map. The background region is chosen from a 3D uniform region (10 × 7 × 9) (Figure 2B). Since the known SBR value for the TRODAT phantom is 9, the SBR error can be calculated as the absolute difference between the estimated SBR value and the ground truth for 40 iterations.

In noisy situations, the coefficient of variation (COV) is measured over a 3D uniform region (10 × 7 × 9) (Figure 2) for both ^99m^Tc-HMPAO and ^99m^Tc-TRODAT, to evaluate the noise performance on the noisy reconstructed images (Eq. 4).


(4) $$COV=\;\frac{\sqrt{\frac{1}{m-1}\sum_{k=1}^{m}{\left(x_{k}-{\bar{x}}_{k}\right)}^{2}}}{{\bar{x}}_{k}}$$

where m is the number of voxels (*m* = 630) in the 3D uniform volume of interest, ${\bar{x}}_{k}$ is the mean voxel value of the VOI, *x*_*k*_ is the voxel value in the noisy reconstructed images, and *k* is the voxel index.

Image profiles are assessed on the reconstructed images of the Derenzo hot-rod phantom (Figure 2C) and star phantom (Figure 2D).

## 3. Results

### 3.1. Analytical simulation

**Figure 4:**
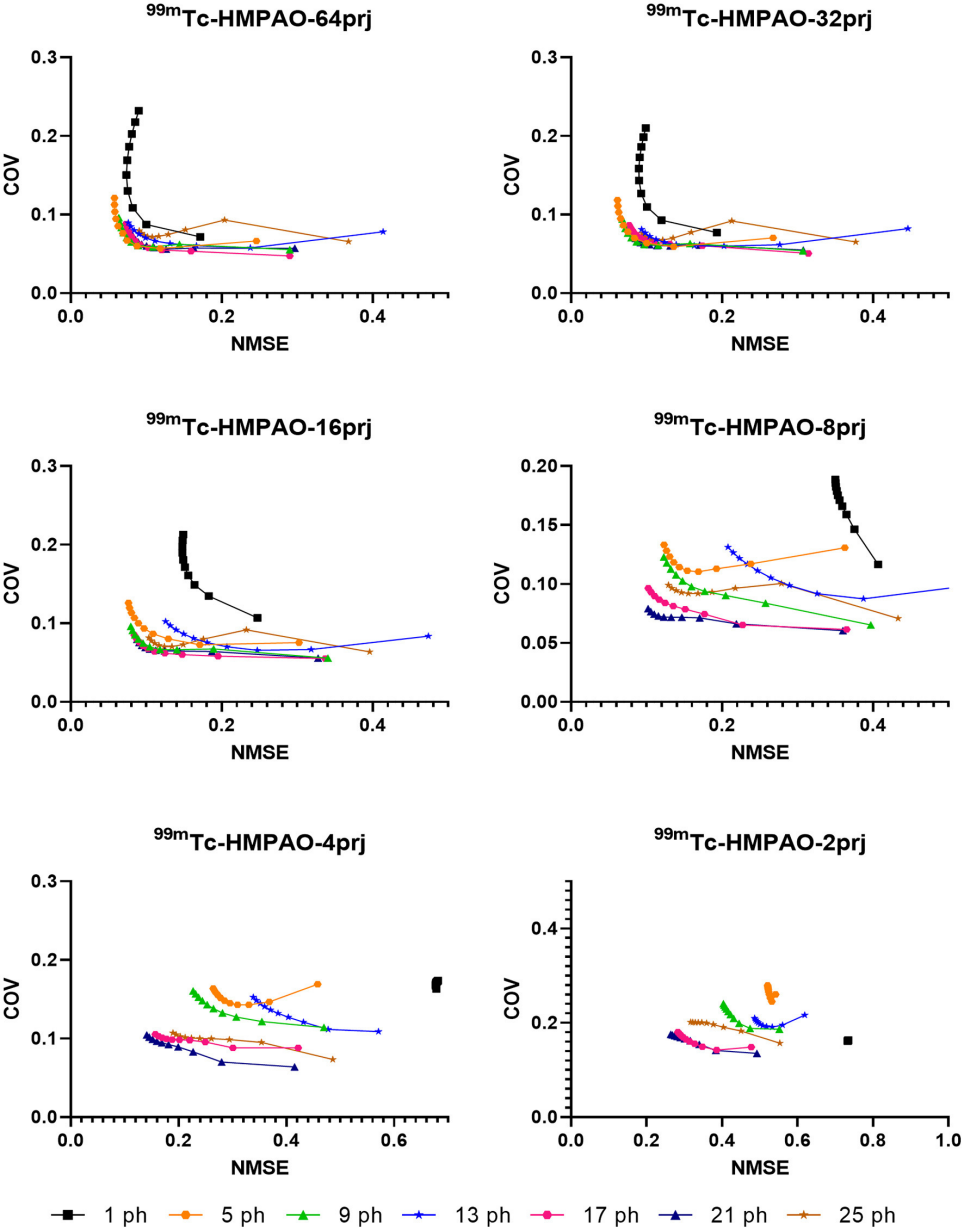
NMSE-COV trade-off for a selected number of pinholes and projections for HMPAO.

**Figure 5:**
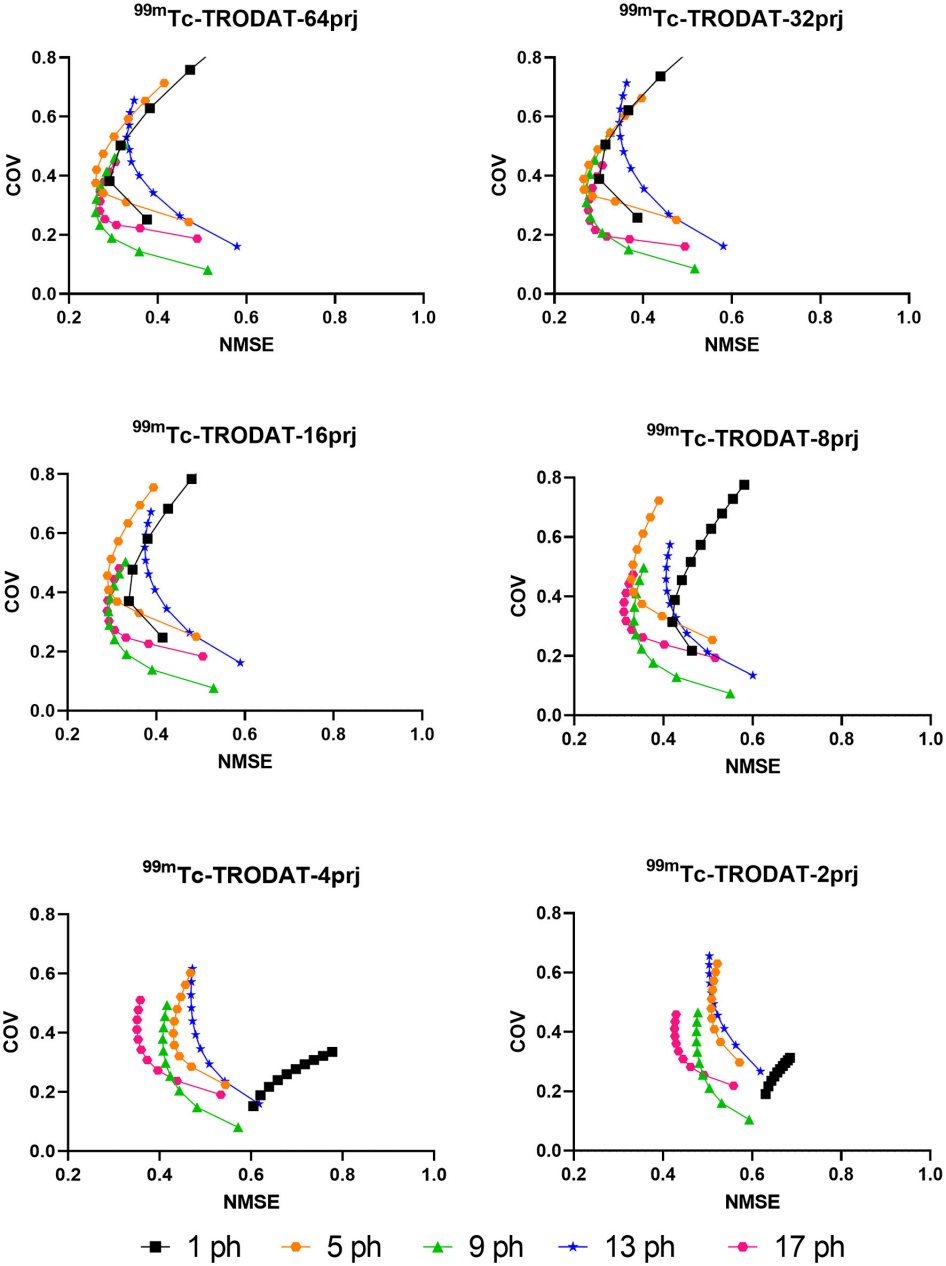
NMSE-COV trade-off of striatum region for a selected number of pinholes and projections for TRODAT.

**Figure 6:**
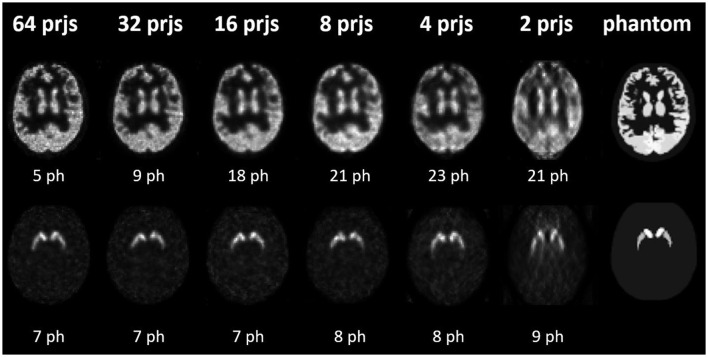
Sample noisy reconstructed images for different numbers of projections with an optimum number of pinholes (ph) having the lowest NMSE values for HMPAO **(top row)** and TRODAT **(bottom row)**.

**Figure 7:**
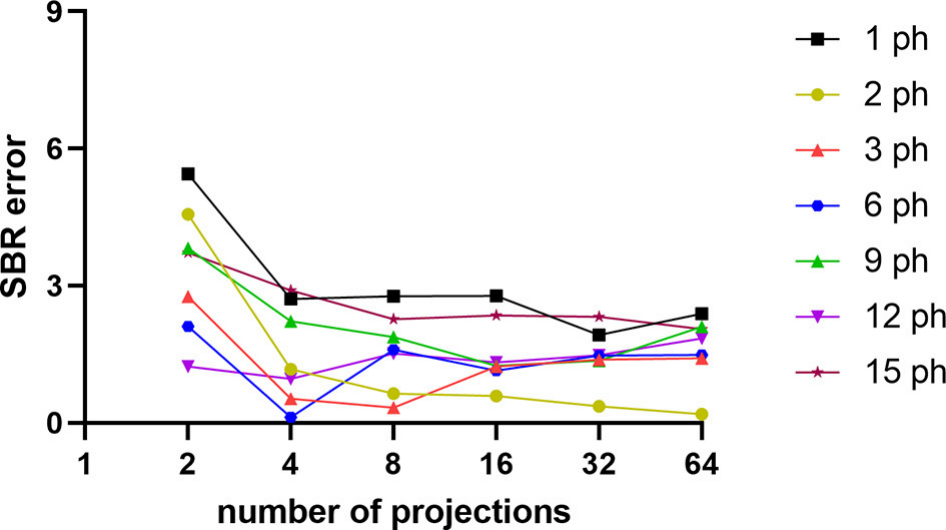
SBR errors for different numbers of pinholes and projections for TRODAT with 40 iterations.

Sample NMSE-COV results of noisy reconstructed images are shown in Figures 4, 5 for HMPAO and TRODAT, respectively. For HMPAO, 5, 9, 18, 21, 23, and 21 pinholes provide the best trade-off for 64, 32, 16, 8, 4, and 2 projections, respectively. For TRODAT, 7, 7, 7, 8, 8, and 9 pinholes provide the best trade-off for 64, 32, 16, 8, 4, and 2 projections respectively. A minimum of 32 projections and 16 projections are required for HMPAO and TRODAT, respectively, to provide a similar NMSE-COV trade-off for 64 projections. Figure 6 shows the sample noisy reconstructed images for different numbers of projections with an optimum number of pinholes for HMPAO and TRODAT. The noise-free results are presented in Supplementary Figures S1, S2. The SBR error results of the TRODAT with 40 iterations are shown in Figure 7. It shows that 2, 2, 2, 3, 6, and 12 pinholes provide the best results for 64, 32, 16, 8, 4, and 2 projections, respectively.

Our results show that NMSE values fluctuate along with the change in detector positions particularly for 2 and 4 projections. L-mode generally leads to lower NMSE values than H-mode for 2 projections. For 4 projections, L-mode with an 80° rotation angle collimator is a better acquisition strategy for HMPAO, while L-mode with a 20° rotation angle is more suitable for TRODAT. For the 6 projections, the differences in NMSE values between various acquisition positions are negligible. The detailed NMSE result of noise-free reconstructed images for different detector positions of 2, 4, and 6 projection views are shown in Supplementary Figure S3 for HMPAO and TRODAT, respectively.

### 3.2. Monte Carlo simulation

**Figure 8:**
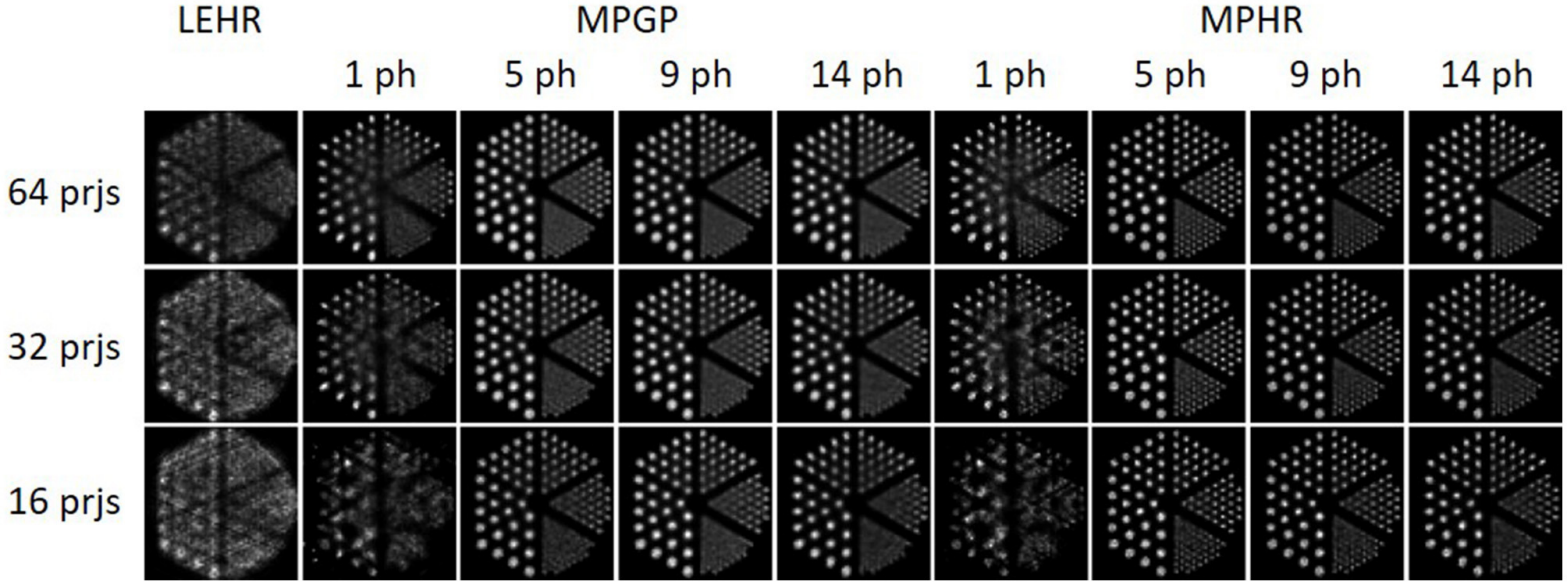
The noise-free reconstructed images of LEHR and 1, 5, 9, and 14 pinholes (ph) for MPGP and MPHR with 16, 32, and 64 projections for the Derenzo hot-rod phantom.

Figure 8 displays sample reconstructed images obtained using MPGP and MPHR. The images show different collimator configurations, including LEHR and 1, 5, 9, and 14 pinholes for comparative analysis. The corresponding profiles are shown in Supplementary Figures S4, S5. MPH collimator provides a large improvement in sampling over the single pinhole collimator and LEHR, especially for fewer projections. MPH could resolve similar sizes of rods for 16–64 projections. The minimum rod diameter that could be resolved is 7.9 mm for MPGP with more than 16 projections and 4.8 mm for MPHR with more than 32 projections. In this case, 9-pinhole and 5-pinhole performed better in MPGP and MPHR, respectively, especially for fewer projections.

**Figure 9:**
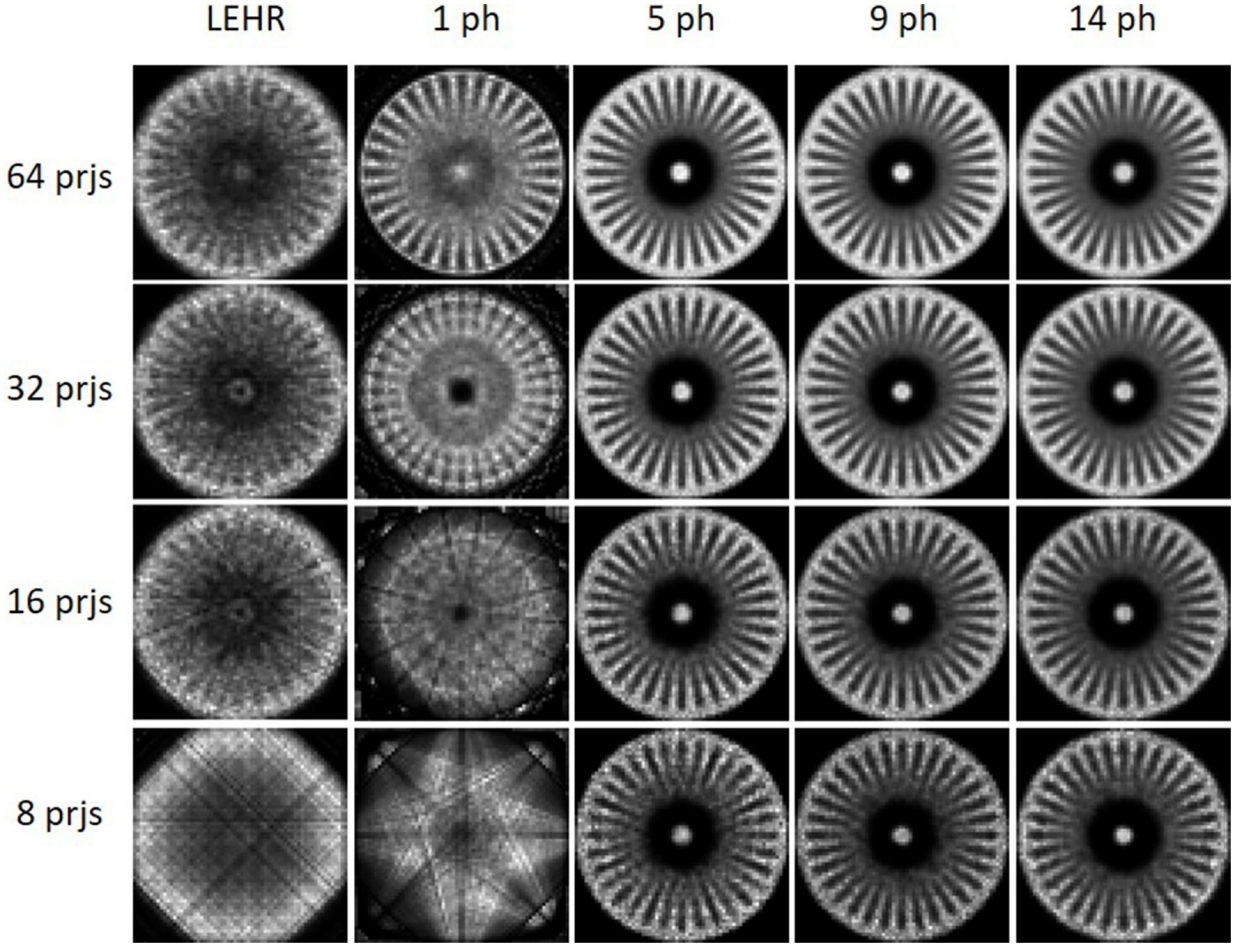
The reconstructed images of LEHR and 1, 5, 9, and 14 pinholes (ph, MPGP) with 8, 16, 32, and 64 projections of the cold star phantom.

The results of the cold star phantom simulations are shown in Figure 9. The corresponding profiles are shown in Supplementary Figure S6. The angular sampling performance is substantially worse for single pinhole and LEHR as compared with MPH. The more projections, the better the angular sampling. In general, the performance of 9-pinhole is the best compared with others. All MPH with different numbers of pinholes can provide enough angular sampling with 32 and 64 projections.

## 4. Discussion

For MPH, multiple magnified or minified projections utilize the whole detector. It improves the system sensitivity and axial and angular samplings (20, 42) as compared with single pinhole or parallel hole collimators which could lead to reduced injection dose or shortened acquisition time for brain SPECT. According to Toossi et al. (43), the smallest hot rods that LEHR could observe are ~9.5–12.7 mm. From Figure 8, one can easily resolve the hot rods with a diameter of 6.4 mm and even 4.8 mm for more projection views when using MPH collimators. Our Monte Carlo results modeling all physics degradations also indicate that MPH collimators are always better than single pinhole and LEHR collimator in terms of sampling, especially for fewer projections. Star phantom simulations (Figure 9) confirm that both numbers of projections and pinholes improve the angular sampling; yet, the differences between 32 and 64 projections for all numbers of pinholes for the MPH collimator are small. These findings align with the results from the study by Zeraatkar et al. (44), who investigated the AdaptiSPECT-C system equipped with 23 detector heads, each comprising a single pinhole aperture. They found that a 2-position increase in angular sampling provided significant benefits, while a 4-position increase was unnecessary. Similarly, Chen et al. (28) evaluated the sampling in G-SPECT, a full-ring focusing MPH SPECT system equipped with 54 focusing pinholes. Their findings indicated that the system performed similarly when using more bed positions as compared with the lowest number of bed positions, i.e., 4. Both studies found that once a sufficient number of samplings were achieved, further increases in sampling did not noticeably impact the performance of the system. Both of the studies utilized full-ring SPECT systems, where angular sampling was less of a concern. However, our study was based on a standard dual-head system, and the influence of angular sampling was more prominent. Our results showed that fewer angular positions required more pinholes to achieve the required angular sampling, which was not described in their studies.

In realistic noisy simulations (Figures 4–6), along with the decrease in the projections, both two tracer distributions required more pinholes to achieve better NMSE-COV trade-offs and SBR values as expected. However, increased projection overlapping and further minimizing the number of projections from excessive pinholes may lead to compromised image quality, as demonstrated in this study, that a 9-pinhole provides better performance as compared with a 15-pinhole for 2 to 6 projections. Additionally, in clinical practice, patient positioning would be easier with a reduced number of pinholes to reduce truncation and projection overlapping. Meanwhile, a more complex activity distribution, i.e., HMPAO, will need more pinholes and projection views for sufficient angular sampling as compared with that of TRODAT. Thus, 32 projection views combined with 9 pinholes would be a good acquisition strategy for HMPAO, while 16 projections combined with 7 pinholes would be a good acquisition strategy for TRODAT. Meanwhile, the number of pinholes also has a greater influence on fewer projections. The optimal number of pinholes is less affected by the change in projection numbers for a smaller VOI as expected, i.e., striatum in TRODAT.

There are limited studies investigating detector positions for MPH brain SPECT, particularly within the inherent geometric constraints of a conventional dual-head scanner. Könik et al. (30) preliminarily evaluated the impact of different angular views on MPH SPECT imaging, while this study provided a more thorough investigation for different pinhole and projection numbers and evaluated the effect of detector positions of a conventional dual-head SPECT system for a limited number of projections. Though a full ring system is expected to provide the best dynamic SPECT performance, Huh et al. (45) proposed a 4D spatiotemporal reconstruction method for dynamic sequences of separate respiratory and cardiac phases, utilizing a conventional dual-head SPECT camera with detector rotations. Fewer angular views which lead to fewer detector movements may be advantageous for potential dynamic brain acquisition. We evaluate the influence of different detector positions on MPH brain SPECT with limited angular views (Figure 3, Supplementary Figure S3). In general, two projections provide undiagnostic image quality, regardless of the number of pinholes and detector positions. For four projections, L-mode with an 80° rotation angle is a better acquisition strategy for HMPAO, while L-mode with a 20° rotation angle is better for TRODAT when the starting angle is 0°.

One limitation of this study is that only normal brain phantoms were used for the simulation. For clinical diagnosis of brain diseases, such as AD and PD, a population phantom with asymmetrical striatum or cerebral perfusion with varying levels of defects would be beneficial for a more realistic assessment of the proposed MPH performance (46). The state-of-the-art 360° CZT SPECT system, equipped with parallel-hole collimators, demonstrates excellent spatial resolution and sensitivity in brain imaging. According to the study mentioned in the reference (47), such a system achieves similar spatial resolution (6.4 mm cold rods resolved) and nearly three times sensitivity (0.116%) as compared with our dual-head MPH system (e.g., 9 PH MPGP with 6.4 mm hot rods resolved and a 0.0372% sensitivity). Given the inherent geometric limitations of current systems, we anticipate that the performance of the proposed dual-head MPH SPECT would be inferior to the 360° CZT SPECT, particularly for dynamic studies. Full correction methods, e.g., attenuation, scatter, and geometric-collimator detector response, are yet to be implemented in the MPH reconstruction, to enhance the image quality; smaller hot rods could possibly be retrieved in the Monte Carlo simulations. We also acknowledge that GATE would provide a more realistic simulation setting for the clinical distribution evaluations. However, due to the practical limitations in computational resources and the research purpose of investigating angular sampling effects of MPH in different projection numbers, we utilize a combination of GATE and analytic simulations in this study. The GATE findings offered additional insights into analytical simulations. A performance comparison study based on physical experiments or Monte Carlo simulation is warranted, but it is beyond the scope of this study. Further studies are warranted to evaluate the improvements of the MPH collimator as compared with the fan-beam collimator, which is also common for the clinic brain SPECT practice.

## 5. Conclusion

In this study, we evaluate the performance of the MPH collimator for various projection views for brain SPECT. Our newly designed MPH collimator offers a spatial resolution of 6.4 mm and 2.2–4.2 times the sensitivity compared with the LEHR collimator for MPGP. For MPHR, it provides a spatial resolution of 4.8 mm and 0.7–1.5 times the sensitivity compared with the LEHR collimator. The number of pinholes affects the performance of the MPH collimator, especially when the projection views become sparse. More pinholes are needed for fewer projections to provide better angular sampling in MPH, especially for complex activity distributions. Our results showed that 32 projection views combined with 9 pinholes would be a good acquisition strategy for HMPAO, while 16 projections combined with 7 pinholes would be a good acquisition strategy for TRODAT. Detector positions substantially affect the image quality for MPH SPECT for 2 and 4 angular views, while L-mode acquisition is slightly superior to H-mode. MPH collimators exhibited improved spatial resolution and angular sampling compared with both LEHR and single pinhole collimators.

## Data availability statement

The raw data supporting the conclusions of this article will be made available by the authors, without undue reservation.

## Author contributions

WH and GM were the primary authors of the manuscript. WH was mainly responsible for data processing and analysis. GM was responsible for data interpretation and study integration. All authors contributed to the article and approved the submitted version.
