# Supplementary material for: Multi-pinhole collimator design in different numbers of projections for brain SPECT
Source: Front Med (Lausanne). 2023 Sep 28;10:1211726. doi: 10.3389/fmed.2023.1211726 (PMC10568733; doi:10.3389/fmed.2023.1211726)
Supplement: Supplementary file 1 [file Data_Sheet_1.docx]

**Supplementary data**

Table S1. Specific parameters for MPH collimators with different number of pinholes.

| Number of pinholes | | 1 | 2 | 3 | 4 | 5 | 6 | 7 | 8 | 9 | 10 | 11 | 12 | 13 |
| --- | --- | --- | --- | --- | --- | --- | --- | --- | --- | --- | --- | --- | --- | --- |
| Collimator to CFOV distance (cm) | | 31.64 | 29.35 | 29.25 | 29.24 | 29.42 | 29.26 | 29.35 | 29.40 | 29.41 | 29.29 | 29.37 | 29.24 | 29.30 |
| Focal length (cm) | | 13.50 | 13.90 | 14.70 | 15.10 | 15.50 | 15.80 | 16.10 | 16.40 | 16.70 | 16.80 | 12.37 | 12.14 | 12.00 |
| Pinhole acceptance angle (°) | | 95.59 | 92.01 | 85.73 | 82.94 | 80.36 | 78.53 | 76.80 | 75.14 | 73.57 | 73.06 | 72.06 | 71.58 | 70.62 |
| Magnification factor | | 1.34 | 1.11 | 0.99 | 0.94 | 0.90 | 0.85 | 0.82 | 0.79 | 0.76 | 0.74 | 0.73 | 0.71 | 0.69 |
| Physical pinhole diameter (cm) | MPGP | 0.65 | 0.59 | 0.56 | 0.54 | 0.53 | 0.51 | 0.50 | 0.49 | 0.47 | 0.46 | 0.46 | 0.45 | 0.44 |
|  | MPHR | 0.42 | 0.37 | 0.35 | 0.33 | 0.32 | 0.31 | 0.30 | 0.29 | 0.28 | 0.27 | 0.27 | 0.26 | 0.25 |
| Total sensitivity (%) | MPGP | 0.016 | 0.022 | 0.026 | 0.029 | 0.032 | 0.034 | 0.035 | 0.036 | 0.037 | 0.039 | 0.036 | 0.040 | 0.042 |
|  | MPHR | 0.007 | 0.009 | 0.011 | 0.012 | 0.013 | 0.013 | 0.013 | 0.014 | 0.014 | 0.014 | 0.014 | 0.015 | 0.015 |

| Number of pinholes | | 14 | 15 | 16 | 17 | 18 | 19 | 20 | 21 | 22 | 23 | 24 | 25 |
| --- | --- | --- | --- | --- | --- | --- | --- | --- | --- | --- | --- | --- | --- |
| Collimator to CFOV distance (cm) | | 29.35 | 29.23 | 29.22 | 29.21 | 29.37 | 29.32 | 29.26 | 29.37 | 29.24 | 29.30 | 29.35 | 29.23 |
| Focal length (cm) | | 11.85 | 11.23 | 11.02 | 10.81 | 10.67 | 10.42 | 10.26 | 10.17 | 10.08 | 9.90 | 9.79 | 9.69 |
| Pinhole acceptance angle (°) | | 69.70 | 67.50 | 66.66 | 65.84 | 64.66 | 63.89 | 63.51 | 72.06 | 71.58 | 70.62 | 69.70 | 67.50 |
| Magnification factor | | 0.68 | 0.62 | 0.61 | 0.59 | 0.57 | 0.55 | 0.54 | 0.53 | 0.53 | 0.51 | 0.50 | 0.49 |
| Physical pinhole diameter (cm) | MPGP | 0.43 | 0.40 | 0.39 | 0.38 | 0.37 | 0.36 | 0.35 | 0.35 | 0.34 | 0.34 | 0.33 | 0.32 |
|  | MPHR | 0.25 | 0.22 | 0.21 | 0.20 | 0.20 | 0.18 | 0.18 | 0.17 | 0.17 | 0.16 | 0.15 | 0.14 |
| Total sensitivity (%) | MPGP | 0.041 | 0.036 | 0.036 | 0.035 | 0.033 | 0.033 | 0.032 | 0.034 | 0.032 | 0.032 | 0.032 | 0.033 |
|  | MPHR | 0.014 | 0.012 | 0.012 | 0.011 | 0.010 | 0.009 | 0.009 | 0.009 | 0.009 | 0.008 | 0.008 | 0.007 |

Table S2. Detector position configurations for 2, 4 and 6 angular views on both HMPAO and TRODAT distributions using analytical simulations.

| Projection number | Acquisition strategy | Detector position angle ( ˚) |
| --- | --- | --- |
| 2 | H-mode (stationary) | (0, 180); (20, 200); (40, 220); (60, 240); (80, 260); (100, 280); (120, 300); (140, 320); (160, 340) |
|  | L-mode (stationary) | (0, 90); (20, 110); (40, 130); (60, 150); (80, 170); (100, 190); (120, 210); (140, 230); (160, 250); (180, 270); (200, 290); (220, 310); (240, 330); (260, 350); (280, 10); (300, 30); (320, 50); (340,70) |
| 4 | H-mode (1 rotation) | (0, 10, 180, 190); (0, 20, 180, 200); (0, 30, 180,210); (0, 40, 180, 220); (0, 50, 180, 230); (0, 60, 180, 240); (0, 70, 180, 250); (0, 80, 180, 260); (0, 90, 180, 270); (0, 100, 180, 280); (0, 110, 180, 290); (0, 120, 180, 300); (0, 130, 180, 310); (0, 140, 180, 320); (0, 150, 180, 330); (0, 160, 180, 340); (0, 170, 180, 350) |
|  | L-mode (1 rotation) | (0, 10, 90, 100); (0, 20, 90, 110); (0, 30, 90, 120); (0, 40, 90, 130); (0, 50, 90, 140); (0, 60, 90, 150); (0, 70, 90, 160); (0, 80, 90, 170); (0, 90, 90, 180); (0, 90, 100, 190); (0, 90, 110, 200); (0, 90, 120, 210); (0, 90, 130, 220); (0, 90, 140, 230); (0, 90, 150, 240); (0, 90, 160, 250); (0, 90, 170, 260); (0, 90, 180, 270) |
| 6 | H-mode (2 rotations) | (0, 60, 120, 180, 240, 300); (10, 70, 130, 190, 250, 310); (20, 80, 140, 200, 260, 320); (30, 90, 150, 210, 270, 330); (40, 100, 160, 220, 280, 340); (50, 110, 170, 230, 290, 350) |
|  | L-mode (2 rotations) | (0, 90, 120, 210, 240, 330); (10, 100, 130, 220, 250, 340); (20, 110, 140, 230, 260, 350); (30, 120, 150, 240, 270, 0); (40, 130, 160, 250, 280, 10); (50, 140, 170, 260, 290, 20) |


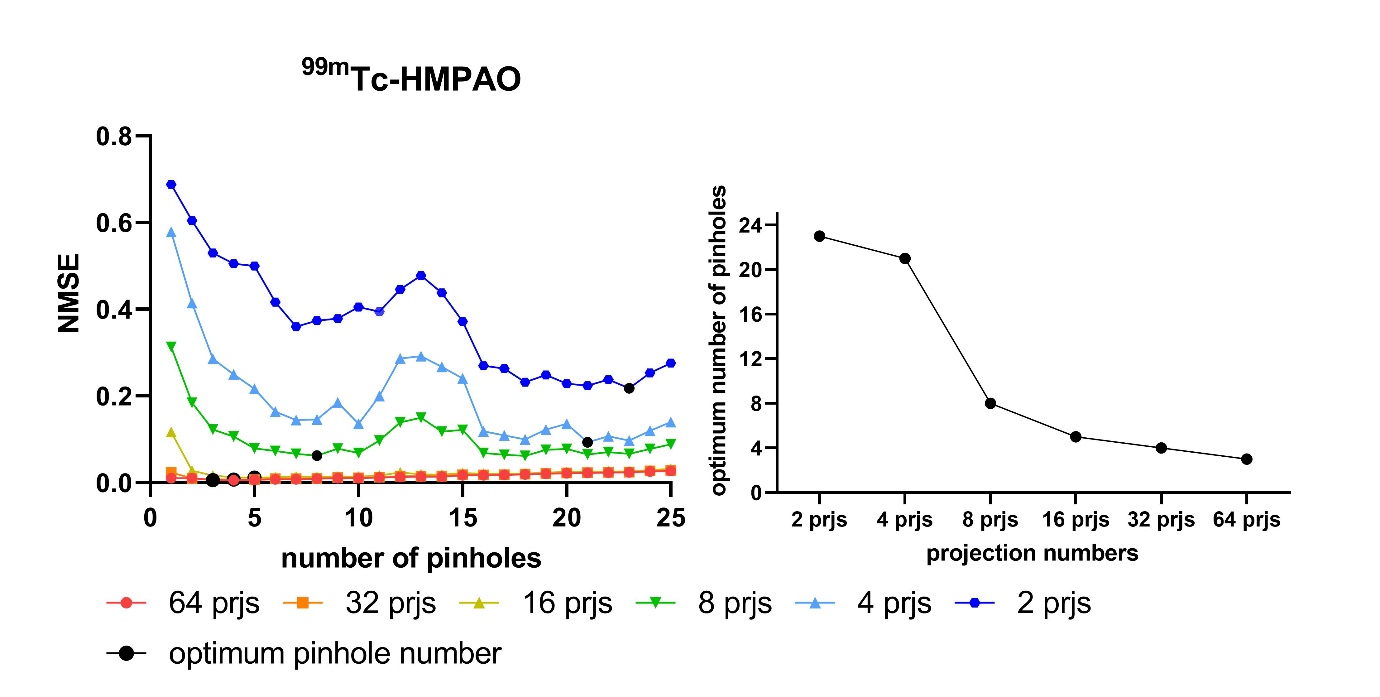


(a)


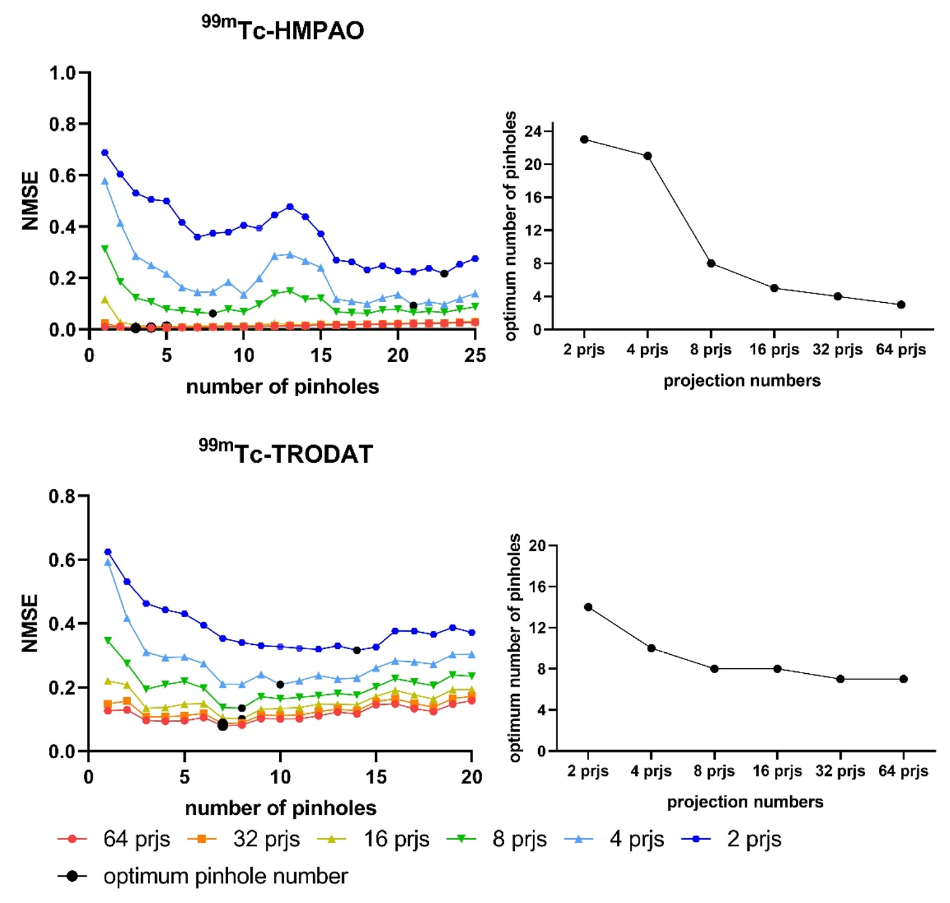


(b)

Figure S1. NMSE results of noise-free reconstructed images for (left) different pinhole and projections numbers and (right) the optimum pinhole number at each projection number with lowest NMSE values for (a) HMPAO and (b) TRODAT.


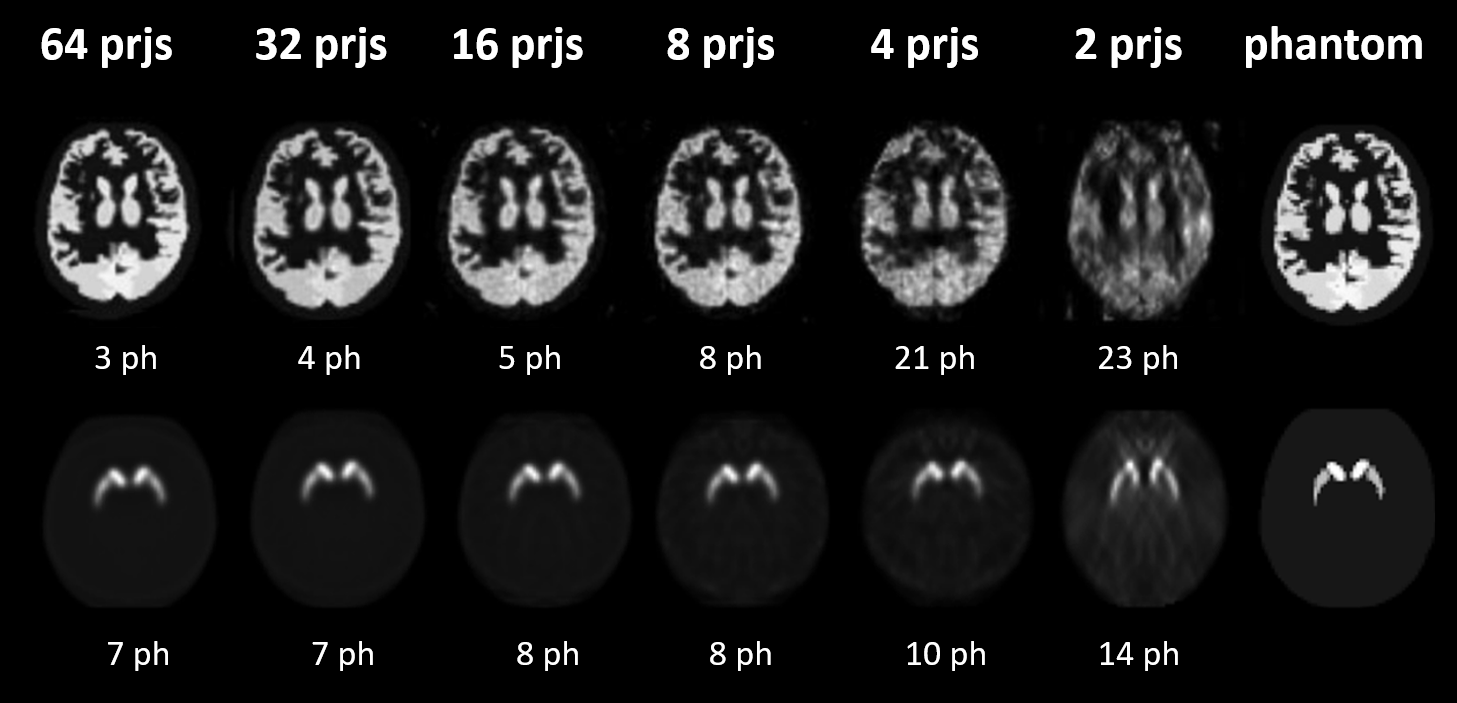


Figure S2. Sample noise-free reconstructed images for different projection numbers with optimum pinhole number (ph) having the lowest NMSE values for HMPAO (top row) and TRODAT (bottom row).


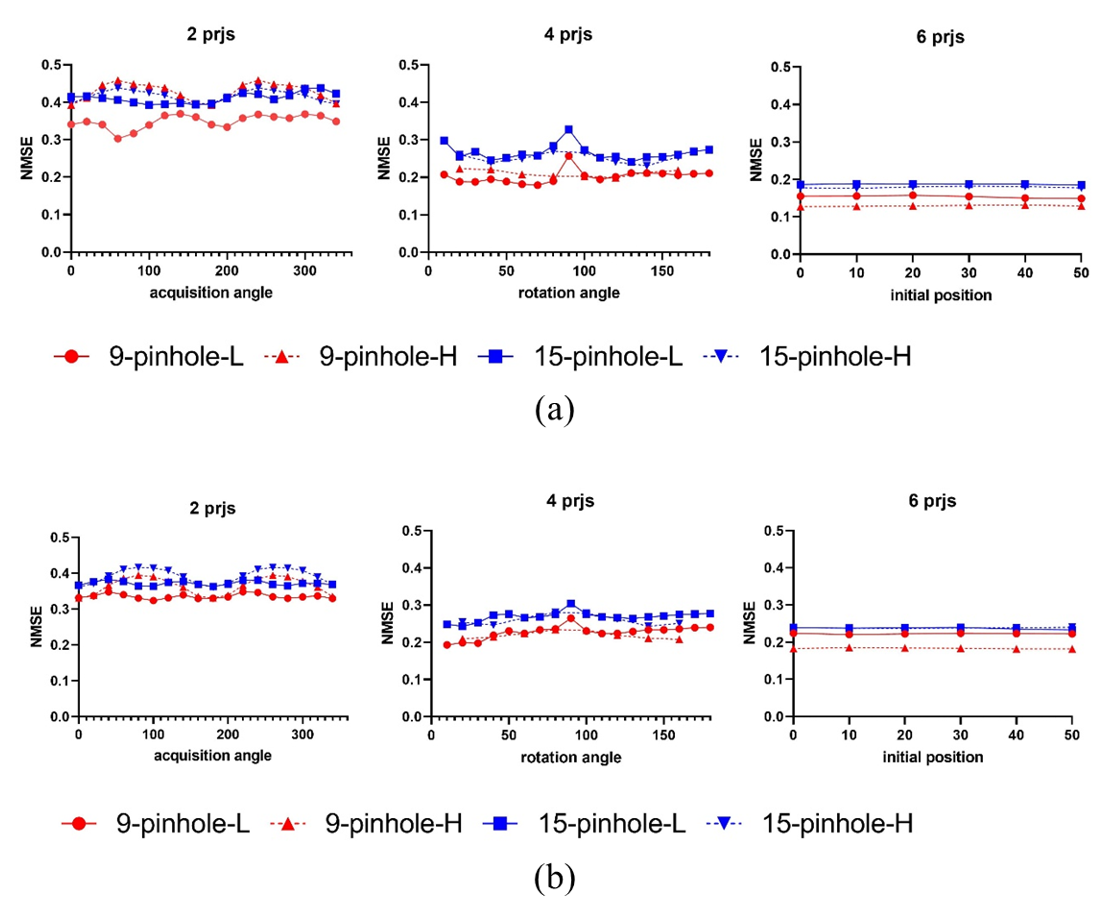


Figure S3. NMSE of 2, 4, 6 projections of H- and L-mode with different detector positions with 9-pinhole and 15-pinhole collimator for (a) HMPAO and (b) TRODAT.


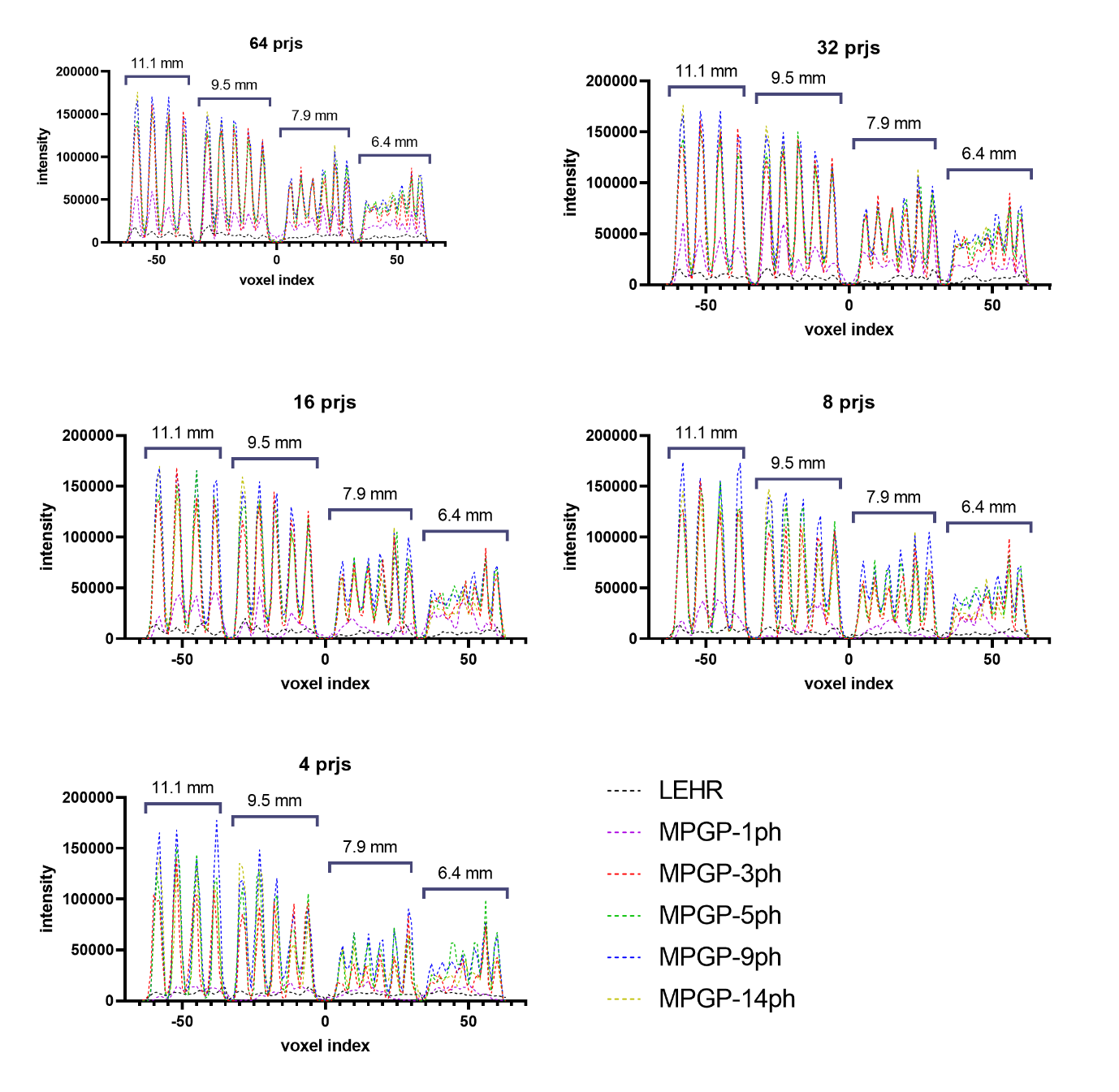
Figure S4. Profiles of the Derenzo hot-rod phantom (labeled in Figure 2c) reconstructed images for MPGP.


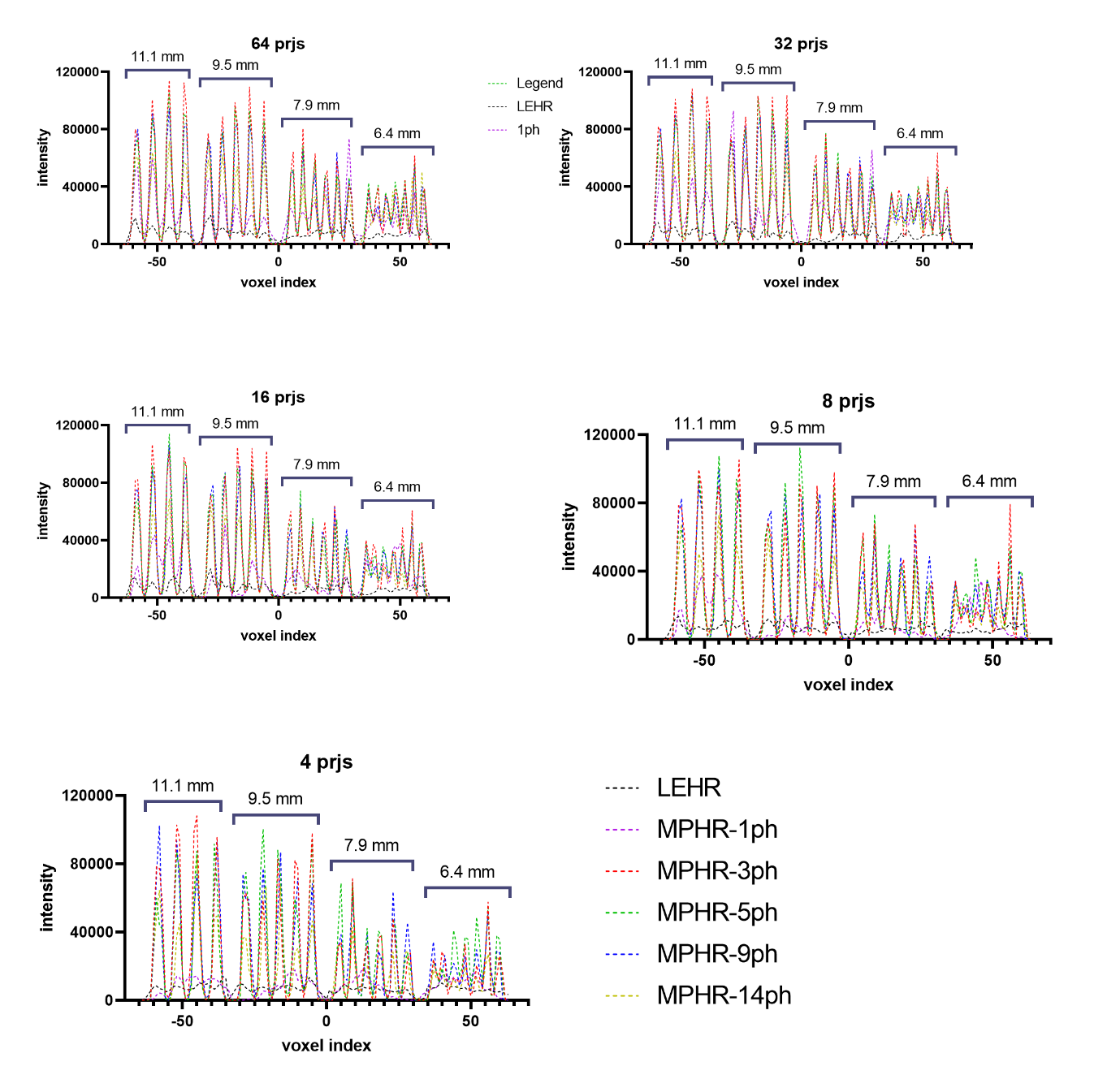
Figure S5. Profiles of Derenzo hot-rod phantom (labeled in Figure 2c) reconstructed images for MPHR.


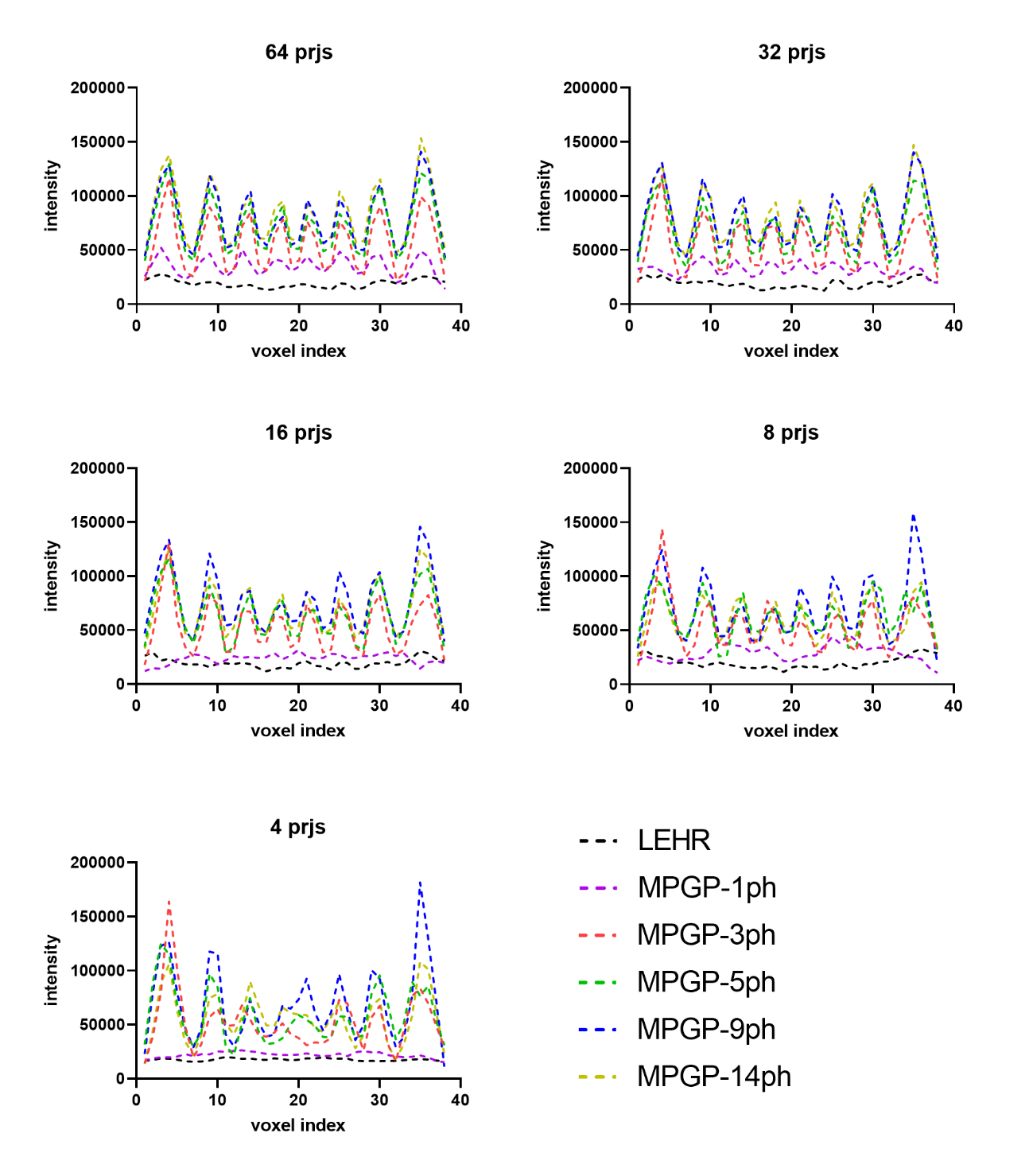
Figure S6. Profiles of star phantom (labeled in Figure 2d) reconstructed images for MPGP.
